# Elevated TERT Expression in TERT-Wildtype Adult Diffuse Gliomas: Histological Evaluation with a Novel TERT-Specific Antibody

**DOI:** 10.1155/2018/7945845

**Published:** 2018-03-05

**Authors:** Kenta Masui, Takashi Komori, Yukinari Kato, Kenkichi Masutomi, Koichi Ichimura, Satoshi Ogasawara, Mika K. Kaneko, Hiroharu Oki, Hiroyoshi Suzuki, Masayuki Nitta, Takashi Maruyama, Yoshihiro Muragaki, Takakazu Kawamata, Tatsuo Sawada, Noriyuki Shibata

**Affiliations:** ^1^Department of Pathology, Tokyo Women's Medical University, Tokyo 162-8666, Japan; ^2^Department of Neuropathology, Tokyo Metropolitan Neurological Hospital, Tokyo 183-0042, Japan; ^3^Department of Antibody Drug Development, Tohoku University Graduate School of Medicine, Miyagi 980-8575, Japan; ^4^Division of Cancer Stem Cell, National Cancer Center Research Institute, Tokyo 104-0045, Japan; ^5^Division of Brain Tumor Translational Research, National Cancer Center Research Institute, Tokyo 104-0045, Japan; ^6^Department of Pathology and Laboratory Medicine, Sendai Medical Center, Miyagi 983-0045, Japan; ^7^Department of Neurosurgery, Tokyo Women's Medical University, Tokyo 162-8666, Japan

## Abstract

Telomerase reverse transcriptase (TERT) is important for the biology of diffuse gliomas.* TERT* promoter mutations are selectively observed among 1p/19q-codeleted oligodendrogliomas and isocitrate dehydrogenase gene-* (IDH-)* wildtype glioblastoma (GBM). However, TERT transcripts range widely in various cancers including gliomas, and TERT protein expression has been rarely investigated thus far. It would be thus critical to examine the expression level of TERT in tumors in addition to its mutational status, and sensitive and specific methods are urgently needed to examine TERT protein expression for the assessment of TERT biology in gliomas. Using our newly developed TERT-specific monoclonal antibody (TMab-6) applicable to human tissue, we found an unexpected increase in TERT expression in* TERT*-wildtype as well as* TERT*-mutated gliomas and in tumor vasculature. This is the first extensive analysis on the expression of TERT immunoreactivity in human glioma tissue, suggesting that TERT protein expression may be regulated by several mechanisms in addition to its promoter mutation.

## 1. Introduction

Diffuse gliomas in adults are now separated into three comprehensive tumor groups with distinctive prognoses, based on the status of isocitrate dehydrogenase genes 1 and 2* (IDH1/2)* mutations and 1p/19q-codeletion [1]. Recently, hotspot mutations in the telomerase reverse transcriptase gene* (TERT)* promoter (chromosome 5: 1,295,228 C>T and 1,295,250 C>T) have been identified in melanomas [2, 3]. The subsequent investigation in a series of gliomas demonstrated the applicability of the* TERT* promoter mutations in the classification of diffuse gliomas [4], and the selectively high frequency of the* TERT* promoter mutations was observed among 1p/19q-codeleted oligodendrogliomas and* IDH*-wildtype glioblastoma (GBM) [5]. The presence of* TERT* promoter mutations is well-correlated with three distinctive glioma groups [1], suggesting that TERT could be deeply involved in the biology of diffuse gliomas.

Hotspot mutations in the* TERT* promoter (C228T and C250T) are currently recognized as the only event in gliomas to upregulate TERT transcripts, and mRNA of TERT tends to be increased in* TERT*-mutated gliomas compared with normal brain tissue or* TERT*-wildtype gliomas [2, 3, 6]. However, the TERT transcripts range widely in expression in various cancers including gliomas [5, 7], and there may be other potential mechanisms to upregulate TERT mRNA in addition to its promoter mutations. Indeed, in other brain tumors and systemic cancers, epigenetic shifts in the* TERT* promoter regions including DNA methylation and histone modifications were reported to regulate TERT transcripts and reactivation [8–10], necessitating a rethinking of the mechanism to upregulate the expression of TERT.

As an essential component of telomerase, TERT adds telomeric repeats to chromosomal ends using telomerase RNA component (TERC) as a template and maintains telomere length. Telomerase activity had been considered to be maintained in most types of the cancer cells, enabling them to proliferate and survive indefinitely [11]. However, a recent report demonstrated that telomere length is positively correlated with mutations in the alpha thalassemia/mental retardation syndrome X-linked gene* (ATRX)* but not the* TERT* promoter, suggesting that TERT upregulation itself may be essential for glioma biology irrespective of telomere length [12]. It would be thus critical to examine the expression level of TERT protein in diffuse gliomas to clarify the role of* TERT* in their biology in addition to its mRNA level as well as mutational status, but few reports have so far validated the expression of TERT protein in human glioma tissue [7]. Sensitive and specific methods are strongly desired to examine TERT protein expression for the future analyses on the TERT biology in diffuse gliomas.

In this study, we developed a TERT-specific antibody applicable to human tissue and examined the expression of TERT in a series of glioma samples. Although the practical diagnostic utility of the antibody to detect the* TERT*-mutated tumors was expected, TERT immunohistochemistry was not capable of identifying the differences between TERT-mutated gliomas and TERT-nonmutant gliomas. Surprisingly, however, an increase in TERT protein expression was detected across all types of gliomas including tumors with wildtype* TERT*, and the level of TERT expression was different even among the* TERT*-mutant gliomas such as oligodendrogliomas and GBMs. Our study is the first thorough analysis on the expression of TERT protein using a specific TERT monoclonal antibody in human glioma tissue, suggesting that TERT protein expression may be regulated by several mechanisms in addition to its promoter mutation.

## 2. Materials and Methods

### 2.1. Tissue Collection

A total of 41 human glioma tissues and 4 nonneoplastic adult human cerebral tissues from four epileptic patients were obtained at surgery from Tokyo Women's Medical University Hospital and Tokyo Metropolitan Neurological Hospital. The tumor samples included 11 grade II and III astrocytic tumors with* IDH* mutations [diffuse astrocytoma (DA) and anaplastic astrocytoma (AA)], 10 grade II and III oligodendroglial tumors, 11 grade IV* IDH*-wildtype GBMs, and 9* IDH*-wildtype diffuse astrocytomas including tumors histologically diagnosed as diffuse astrocytoma, anaplastic astrocytoma, oligoastrocytoma, and anaplastic oligoastrocytoma which did not show* IDH* mutations, p53 immunopositivity, and the loss of ATRX expression (wtIDH/p53-/ATRX+). Clinical information of the cases was collected from the medical records. All specimens were fixed in 10% buffered formalin and embedded into paraffin sections. Sections were routinely stained with hematoxylin and eosin (H&E), and histopathological diagnoses were made according to the World Health Organization (WHO) 2016 classification of tumors of the nervous system [13–15] and International Society of Neuropathology-Haarlem consensus guidelines [16].

**Figure 1:**
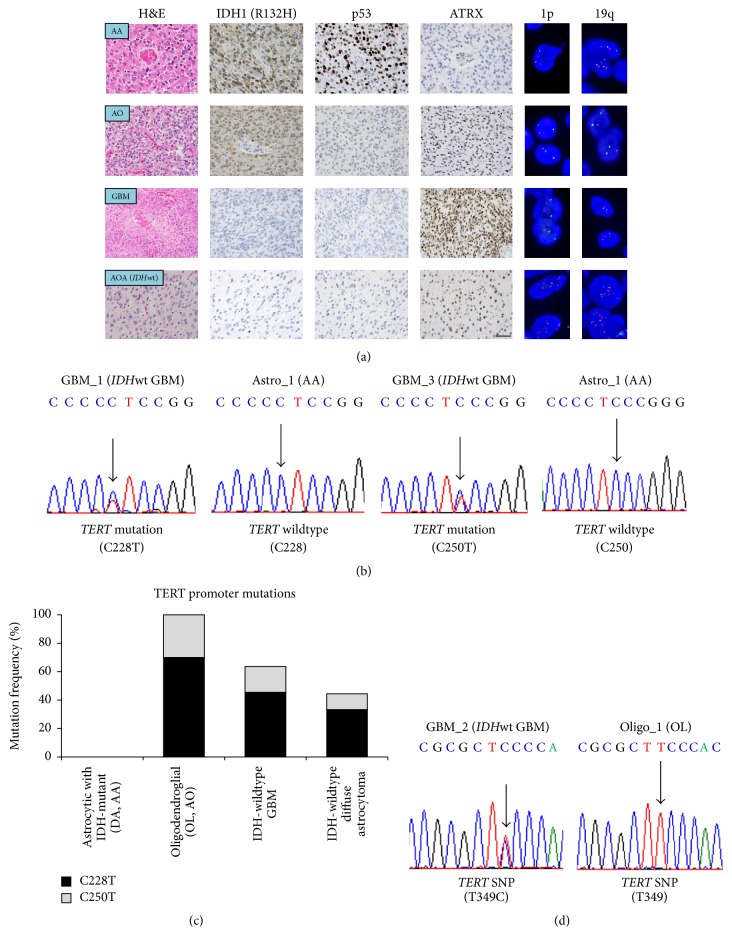
Molecular profile of the glioma cases. (a) Diffuse astrocytic (DA/AA) tumors were characterized by immunopositivity for IDH1 (R132H) and p53, and the loss of immunoreactivity for ATRX. Oligodendroglial tumors demonstrated IDH1 (R132H) immunoreactivity and 1p/19q-codeletion on FISH (red: probes for 1p36 or 19q13, green: probes for the centromere of chromosome 1 or 19). Note that these genotypes of astrocytomas and oligodendrogliomas are mutually exclusive except for* IDH* genes.* IDH*-wildtype GBM tumors did not possess mutations in* IDH* genes. The* IDH*-wildtype diffuse astrocytoma group did not show any molecular genetic aberrations shown above. (b) Mutations at C228 and C250 of the* TERT* promoter region were examined for every case by Sanger sequencing. Representative cases were shown for* TERT* promoter mutations and wildtype* TERT*. (c) Examination of the frequency of* TERT* promoter mutations in each group of gliomas. Oligodendroglial tumors and* IDH*-wildtype GBM possessed high incidence of* TERT* mutations, followed by the* IDH*-wildtype diffuse astrocytoma. No grade II and III astrocytic tumors with IDH-mutant showed* TERT* promoter hotspot mutations. (d) Sanger sequencing results displayed representative cases for* TERT* promoter common SNPs (T349C). The molecular profile of the cases was summarized in Table 1 and Supplementary Table  1. AA, anaplastic astrocytoma; AO, anaplastic oligodendroglioma; AOA, anaplastic oligoastrocytoma; DA, diffuse astrocytoma; GBM, IDH-wildtype glioblastoma; OL, oligodendroglioma; wt, wildtype. Scale bar = 40 *μ*m.

All tumor and control brain samples were subjected to histological diagnosis followed by molecular genetics screening for a more precise/molecular diagnosis (Figure 1(a)) [17]. Tumors in the astrocytoma group (DA/AA) were characterized by detectable immunoreactivity for mutated IDH1 (R132H) and p53 [18] and the loss of immunoreactivity for ATRX [19, 20]. All tumors in the oligodendroglioma group demonstrated mutated IDH1 (R132H) and 1p/19q-codeletion. GBM tumors did not possess mutations in* IDH*, corresponding to “GBM,* IDH*-wildtype” [14, 21]. The tumors in the* IDH*-wildtype diffuse astrocytoma group did not show any molecular genetic aberrations shown above. The study was approved by the institutional review boards, and the detailed information of the cases was provided in Supplementary Table  1.

### 2.2. DNA Extraction and Sanger Sequencing

Genomic DNA was extracted from formalin-fixed paraffin-embedded (FFPE) tissue of the solid tumor regions confirmed on H&E sections, using QIAamp DNA FFPE Tissue Kit (QIAGEN, Venlo, The Netherlands), followed by the purification with NucleoSpin gDNA Clean-up Kit (MACHEREY-NAGEL, Dusseldorf, Germany), according to the manufacturer's instructions. Sequences of the primer sets for polymerase chain reaction (PCR) were provided in Supplementary Table 2. PCR was performed using AmpliTaq Gold 360 DNA Polymerase and GC enhancer (Thermo Fisher Scientific, Waltham, MA). Sequencing was carried out by Eurofins Genomics (Tokyo, Japan) with each forward PCR primer as a sequencing primer.

### 2.3. Molecular Diagnostics

The status of IDH1 (R132H), p53, and ATRX was analyzed by immunohistochemistry using the automated immunostaining processor Histostainer (Nichirei Biosciences, Tokyo, Japan). The antibodies used include anti-IDH1 (R132H) (DIA-H09; Dianova, Hamburg, Germany), anti-p53 (DO-7; Nichirei), and anti-ATRX (HPA001906; Sigma-Aldrich, St. Louis, MO). We judged the tumor as p53-positive when >50% of the tumor nuclei displayed the strong immunoreactivity of p53, which could be corroborated by the loss of immunoreactivity for ATRX (inactivating mutation) and 1p/19q-nondeletion in the astrocytic tumors [19, 20, 22]. The status of chromosomes 1p and 19q was examined by fluorescence in situ hybridization (FISH) with 1p36 and 19q13 probes (Vysis; Abbott Molecular, Abbott Park, IL), and signal ratios were calculated according to the previous report [23]. Two reported hot spot mutations (C228T and C250T) in a* TERT* promoter region were analyzed by Sanger sequencing. The hotspot mutations at codon 132 of* IDH1* and codon 172 of* IDH2* were also screened by Sanger sequencing if the cases did not show immunoreactivity for mutated IDH1 (R132H) [24, 25].

### 2.4. Cell Lines

U87 malignant glioma cell lines are a kind gift from Dr. Paul Mischel laboratory (Ludwig Institute for Cancer Research, San Diego). Cells were cultured in DMEM supplemented with 10% FBS (Omega Scientific, Tarzana, CA) in a humidified 5% CO2 incubator at 37°C. Analyses with immunocytochemistry and Sanger sequencing were performed using the cell lines as described below.

### 2.5. Development of TERT-Specific Antibody

Human TERT (hTERT) cDNA (Accession number NM_001193376.1) encoding a specific peptide composed of Glu281-Ala436 amino acid residues was obtained by PCR using a cDNA derived from the LC-AI or HT1080 cell line as a template. The primer set for hTERT was as follows: 5′-AAGGATTTCAGAATTCGAAGCCACCTCTTTGGA-3′ (forward) and 5′-TGCCGTCTCCGAATTCCGCCACAGAGCCCTGGG-3′ (reverse). The hTERT-specific peptide was subcloned into an expression vector, pMAL-c2 (New England Biolabs, Beverly, MA) with MAP tag (GDGMVPPGIEDK) [26], and PA tag (GVAMPGAEDDVV) [27] using In-Fusion PCR cloning kit. Competent* Escherichia coli* (*E*.* coli*) TOP-10 cells (Thermo Fisher Scientific) were transformed with the plasmid, pMAL-c2MAPhPAter/TERTepi. Then, they were cultured overnight at 37°C in LB medium (Thermo Fisher Scientific) containing 100 *μ*g/ml ampicillin (Sigma-Aldrich, St. Louis, MO). Cell pellets were resuspended in phosphate-buffered saline (PBS; nacalai tesque, Kyoto, Japan) with 1% Triton X-100 with 50 *μ*g/ml aprotinin (Sigma-Aldrich). After sonication, the crude extracts were collected by centrifugation (9000 ×g, 30 min, 4°C). The supernatants were loaded onto Amylose resin. The loaded resins were washed extensively with column buffer consisting of 20 mM Tris-HCl (pH 7.4), 200 mM NaCl and 1 mM EDTA, and the fusion proteins were eluted by column buffer with 10 mM maltose.

BALB/c mice were immunized by intraperitoneal (i.p.) injection of the synthetic peptide of hTERT (302–321 amino acids; Sigma-Aldrich) together with Imject Alum (Thermo Fisher Scientific). After several additional immunizations, a booster injection was given i.p. two days before splenocytes were harvested. The splenocytes were fused with P3U1 cells obtained from the American Type Culture Collection (ATCC; Manassas, VA) using PEG1500 (Roche Diagnostics, Indianapolis, IN). The hybridoma cells were grown in Roswell Park Memorial Institute (RPMI) medium with hypoxanthine, aminopterin, and thymidine selection medium supplement (Thermo Fisher Scientific). The culture supernatants were screened using direct enzyme-linked immunosorbent assay (ELISA) for the binding to synthetic peptide and recombinant protein of hTERT purified from* E*.* coli*. The Animal Care and Use Committee of Tohoku University approved the animal experiments about hybridoma production in this study.

### 2.6. Verification of the Specificity of Anti-hTERT Antibodies Using ELISA

Synthetic peptide and recombinant protein of hTERT were immobilized on Nunc Maxisorp 96-well microplates (Thermo Fisher Scientific) at a concentration of 1 *μ*g/ml and 5 *μ*g/ml, respectively, for 30 min. After blocking with 1% bovine serum albumin (BSA) containing 0.05% Tween20 in PBS, the plates were incubated with culture supernatant followed by 1 : 3000 diluted peroxidase-conjugated anti-mouse IgG (Dako, Glostrup, Denmark). The enzymatic reaction was conducted with a 1-Step Ultra TMB-ELISA (Thermo Fisher Scientific). The optical density was measured at 655 nm using an iMark microplate reader (Bio-Rad Laboratories, Berkeley, CA).

### 2.7. Immunoprecipitation-RNA-Dependent RNA Polymerase (IP-RdRP) Assay

1 × 10^7^ cells treated with nocodazole were lysed in 1 ml of lysis buffer A [0.5% NP-40, 20 mM Tris-HCl (pH 7.4) and 150 mM NaCl]. After sonication, cell lysates were cleared of insoluble material by centrifugation at 21,000 ×g at 4°C for 15 min. One milliliter of the lysates was preabsorbed 2 times with 40 *μ*L of Protein L Agarose (Sigma-Aldrich) for 30 min at 4°C. The preabsorbed lysates were mixed with 10 *μ*g of an anti-human TERT monoclonal antibody and 40 *μ*L of Protein L Agarose and incubated overnight at 4°C. The remaining of the IP-RdRP assay was performed as described previously [28, 29].

### 2.8. Immunohistochemistry for TERT

All sections were deparaffinized in xylene and rehydrated in an ethanol gradient. Sections were subjected to antigen retrieval by microwaving in 10 mM citrate buffer (pH 6.0) for 20 min (95°C, 500 W). Endogenous peroxidase activity was quenched with 0.3% H_2_O_2_ in methanol. The sections were then incubated with a primary antibody [TMab-6; mouse IgM, kappa; diluted in 1 : 2000 (0.5 *μ*g/mL)] at room temperature for 90 min. The optimal concentration of the primary antibody was determined by a serial dilution of the antibody. After rinsing, the sections were subjected to the polymer-immunocomplex method using EnvisionTM (Dako) for enhancing. 3,3′-Diaminobenzidine tetrahydrochloride (DAB; Dojindo, Kumamoto, Japan) was used as the chromogen and hematoxylin as the counterstain. To confirm the specificity of the TERT expression state in glioma cells, negative control reaction was performed by omission of the primary antibodies, and preabsorption test of the antibodies was also conducted using the synthetic peptide of hTERT (Sigma-Aldrich) at a final concentration of 0.1 mg/mL. The percentage of TERT-positive cells in each case was semiquantitatively evaluated as 1+ (a few weakly positive cells), 2+ (positive cells < 50%), and 3+ (positive cells ≥ 50%). We also immunostained the glioma samples with the most widely used, commercially available antibody for TERT (sc-7215; Santa Cruz Biotechnology, Santa Cruz, CA), but the staining was rather faint than that by TMab-6 (Supplementary Figure  1). Thus, we evaluated immunohistochemical observations of TERT in all the glioma samples with our newly developed TMab-6.

### 2.9. Western Blotting

Immunoblotting was performed according to a modified version of the previously reported methods [30, 31]. Snap-frozen tissue samples were lysed and homogenized with radioimmunoprecipitation assay (RIPA) lysis buffer [50 mM Tris-HCl, 150 mM NaCl, 1% NP-40, 0.5% sodium deoxycholate, and 0.1% sodium dodecyl sulfate (SDS)] from Boston BioProducts (Boston, MA). Protein concentration of each sample was determined using the BCA kit (Thermo Fisher Scientific) according to the manufacturer's instructions. Equal amounts of protein extracts were separated by electrophoresis on Any kD Mini-PROTEAN TGX Precast Gels (Bio-Rad) and then transferred to a nitrocellulose membrane with Trans-Blot Turbo Transfer System (Bio-Rad). The membrane was probed with the primary antibodies against TERT (sc-7215) and glyceraldehyde phosphate dehydrogenase (GAPDH) (D16H11; Cell Signaling Technology, Danvers, MA), followed by secondary antibodies conjugated to horseradish peroxidase. TMab-6 was not available for the immunoblotting study because TMab-6 was developed specifically for the immunohistochemical purpose and produced nonspecific bands on Western blots. The immunoreactivity was detected with Super Signal West Pico Chemiluminescent Substrate or West Femto Trial kit (Thermo Fisher Scientific). Quantitative densitometry analysis was performed with image analysis software (ImageJ version 1.49, NIH).

### 2.10. Quantitative Reverse Transcription Polymerase Chain Reaction (qRT-PCR) Analysis

Total RNA was extracted by the use of RNeasy Plus Mini Kit (QIAGEN). Firststrand cDNA was synthesized by the use of iScript™ RT Supermix for RT-qPCR (Bio-Rad). Real-time RT-PCR was performed with the SYBR® Premix Ex Taq™ II (Tli RNaseH Plus) (Takara, Kyoto, Japan) on Thermal Cycler Dice Real Time System TP800 (Takara) following the manufacturer's instructions. *β*-Actin was used as an endogenous control. Primer sequences were provided in Supplementary Table  2.

### 2.11. Statistical Analysis

The data were routinely compared between three or more groups with a two-way analysis of variance (ANOVA) followed by post hoc test Bonferroni corrections for TERT expression using the Statistical Package for the Social Sciences software package (Version 16.0; SPSS, Chicago, IL, USA). *p* value below 0.05 was considered statistically significant.

## 3. Results

### 3.1. Molecular Profiling of the Cases

All 41 tumor samples and 4 control brain samples were subjected to histological as well as molecular genetic diagnostics, and mutations at C228 and C250 of the* TERT* promoter region were subsequently examined for every case by Sanger sequencing, successfully detecting these two reported hotspot mutations (Figure 1(b)). As a result, mutations in the* TERT* promoter were found in 21 out of 41 tumor samples (51.2%). Consistent with previous reports [5], oligodendroglial tumors (10 mutations out of 10 tumors: 100%) and* IDH*-wildtype GBM (7 mutations out of 11 tumors: 63.6%) possessed high incidence of* TERT* promoter mutations whereas no astrocytic tumors with* IDH* mutations showed them (Figure 1(c)). Interestingly, some of the* IDH*-wildtype diffuse astrocytomas demonstrated hotspot mutations in the* TERT* promoter (4 mutations out of 9 tumors: 44.4%). In addition to the* TERT* promoter hotspot mutations, we identified a common single nucleotide polymorphism (SNP) (T349C) [32] across all types of gliomas we investigated (Figure 1(d)):* IDH*-mutant astrocytic tumors (6 out of the 11 tumors: 54.5%), oligodendroglial tumors (3 out of the 10 tumors: 30.0%), GBM (10 out of the 11 tumors: 90.9%), and* IDH*-wildtype diffuse astrocytomas (3 out of the 9 tumors: 33.3%). The molecular profile of the cases was summarized in Table 1 and Supplementary Table  1.

### 3.2. Production and Validation of a Novel Anti-TERT Monoclonal Antibody Available for Human Tissue

**Figure 2:**
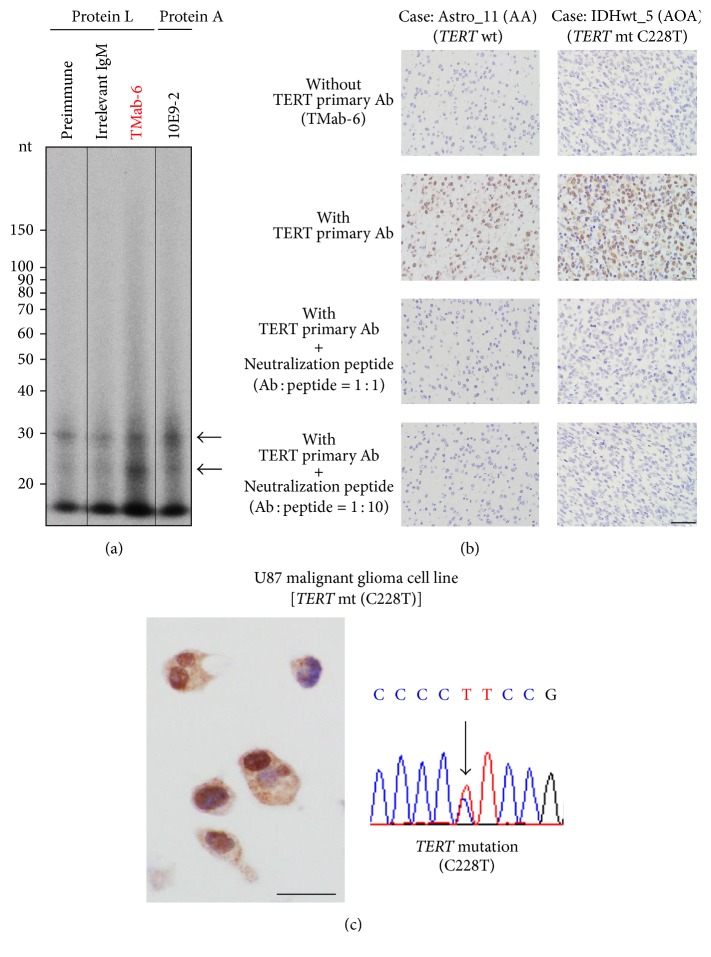
Validation of a newly developed TERT-specific antibody (TMab-6) usable for human glioma tissue. (a) Endogenous TERT was immunoprecipitated with an anti-human TERT mAb (TMab-6) followed by an RNA-dependent RNA polymerase (RdRP) assay using HeLa cells treated with nocodazole. Arrows indicate RdRP products. The preimmune (without antibodies) and irrelevant IgM lanes are a negative control, and the 10E9-2 antibody (MBL, Nagoya, Japan) lane is a positive control. nt, nucleotide. (b) The* IDH*-mutant astrocytoma (anaplastic astrocytoma: AA) and* IDH*-wildtype diffuse astrocytoma (Histologically anaplastic oligoastrocytoma: AOA) cases were analyzed for TERT protein expression. TMab-6 specifically recognized the nuclei of the tumor cells, and immunoreactivity was not detected in the negative control section without the application of TMab-6. An antibody absorption test with synthetic neutralization peptide of TERT further validated the specificity of TMab-6 as an anti-TERT antibody which is applicable to human glioma tissue. (c) TMab-6 Immunostaining of U87 malignant glioma cell lines with a* TERT* hotspot mutation (C228T) showed strong nuclear immunoreactivity for TERT. Ab, antibody; mt, mutation. Scale bar = 40 *μ*m.

In order to evaluate the expression of TERT protein in human glioma tissue, we developed and screened novel anti-hTERT monoclonal antibodies that will be applicable to human tissue sections. We generated about 10,000 hybridomas from two mice and picked up about 150 wells by using ELISA. Next, we screened by using ELISA (synthetic peptide and recombinant protein), western blotting (recombinant protein and GBM cell line lysate), and immunohistochemistry. Some clones were excluded because of nonspecific reactivity by these analyses. Finally, we established nine hybridomas as TMab clones. Among these, TMab-6 showed sensitive reactivity in immunohistochemistry with human tumor tissue of anaplastic oligodendroglioma (IDH-mutant and 1p/19q-codeleted), glioblastoma (IDH-wildtype), and anaplastic astrocytoma (IDH-mutant). In contrast, the other clones did not show sensitive and specific reactivity in immunohistochemistry. We used clone TMab-6 for further studies. Next, we validated the specificity of TMab-6 by immunoprecipitation-based assessment of the enzymatic activity of TERT as well as antibody preabsorption test for tissue sections (Figures 2(a) and 2(b)). Since TMab-6 was IgM clone, we immunoprecipitated endogenous TERT complex by protein L beads from HeLa cells synchronized in mitotic phase by nocodazole [28, 29]. We found that a novel anti-hTERT mAb TMab-6 specifically recognized human TERT protein, and that it could be applied for immunohistochemistry on the human glioma tissue sections. We further confirmed the nuclear staining of TERT by TMab-6 in U87 malignant glioma cell lines (Figure 2(c)) that were previously reported to possess* TERT *hotspot mutations as well as upregulated TERT transcripts [5, 33].

### 3.3. Expression of TERT in Human Glioma Tissue

**Figure 3:**
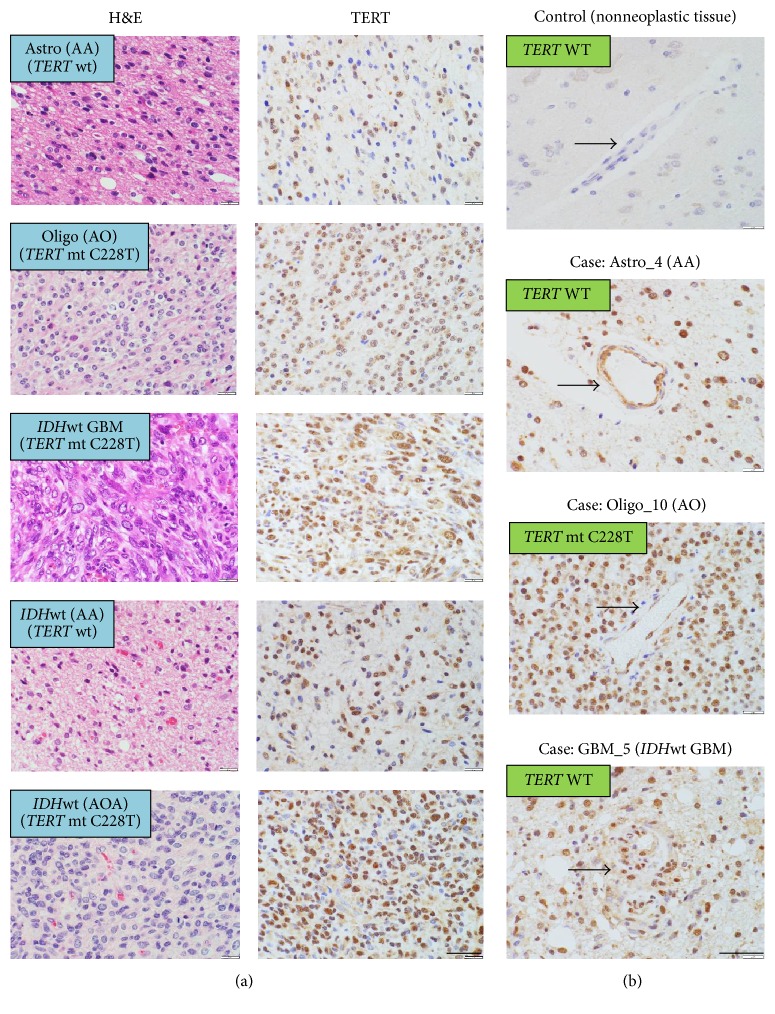
TERT expression in human glioma samples. (a) TMab-6 immunostaining of the glioma samples demonstrated strong nuclear as well as some cytoplasmic staining in tumor cells. Representative tumors were shown in the astrocytic, oligodendroglial,* IDH*-wildtype GBM, and* IDH*-wildtype diffuse astrocytoma groups. Glioma cells in all the cases showed nuclear immunostaining of TERT to a various degree. (b) TMab-6 immunostaining of vascular endothelial cells (arrows) in the nonneoplastic cerebral tissues,* TERT*-wildtype AA,* TERT*-mutated AO, and* TERT*-mutated GBM. AA, anaplastic astrocytoma; AO, anaplastic oligodendroglioma; AOA, anaplastic oligoastrocytoma; GBM,* IDH*-wildtype glioblastoma; mt, mutation; wt/WT, wildtype. Scale bar = 40 *μ*m.

After confirming the specificity of a newly developed anti-TERT monoclonal antibody TMab-6, we performed immunohistochemical staining on sections of nonneoplastic adult human cerebral tissues and all glioma samples including* IDH*-mutated astrocytoma, oligodendroglioma,* IDH*-wildtype GBM, and* IDH*-wildtype diffuse astrocytoma and examined the staining patterns of TERT protein in them. Immunohistochemical observations using TMab-6 demonstrated strong staining in the nucleus and some cytoplasmic staining of the glioma cells (Figure 3(a)), and TERT immunoreactivity was not observed in the normal tissue adjacent to the tumor tissue. Surprisingly, glioma cells in all the cases showed nuclear immunostaining for TERT to various degrees compared with the cells in nonneoplastic tissues (Figure 3(a)) although some intratumoral heterogeneity of the nuclear staining was observed. Some reactive astrocytes in cerebral parenchyma from the cases with hippocampal sclerosis and cortical dysplasia showed nonspecific, weak cytoplasmic staining (Supplementary Figure  2). Interestingly, endothelial cells of the tumor vessels also displayed nuclear immunostaining in both* TERT*-mutated and wildtype tumors, whereas vascular endothelial cells in the nonneoplastic tissues did not show any specific TERT immunoreactivity in their nuclei (Figure 3(b)).

### 3.4. Semiquantitative Evaluation of TERT Expression Associated with TERT Promoter Mutations in Human Gliomas

**Figure 4:**
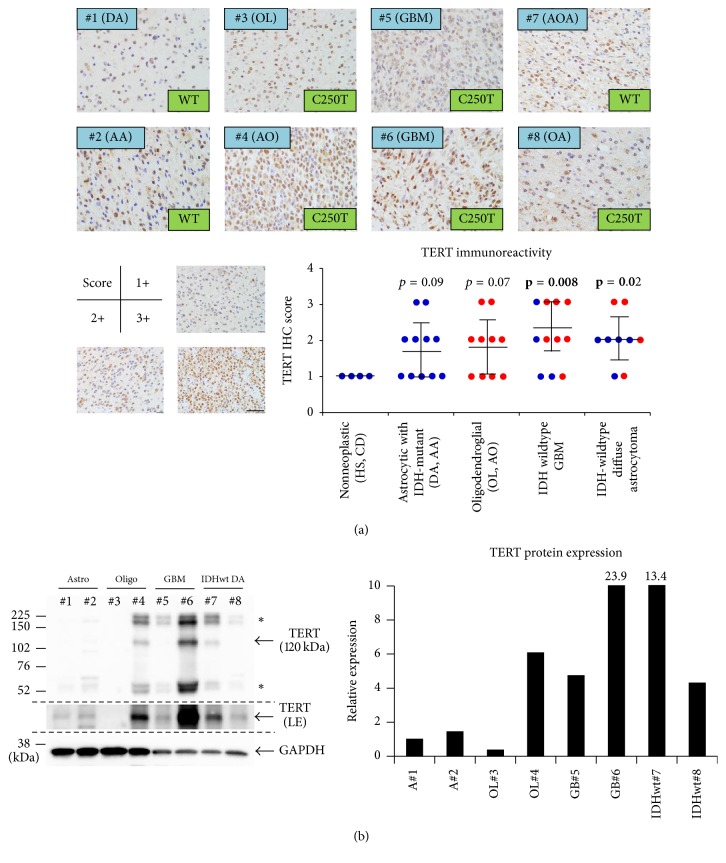
Association of TERT promoter mutations and its immunoreactivity in human gliomas. (a) Immunostaining of TERT by TMab-6 demonstrated that TERT immunoreactivity did not correspond to the mutational status of the* TERT* promoter region across glioma samples. Semiquantitative analysis of the TERT-immunostaining, scoring the staining intensities as 1+ (a few weakly positive cells), 2+ (positive cells < 50%), and 3+ (positive cells ≥ 50%). Blue dots represent* TERT*-wildtype cases and red denotes* TERT*-mutated cases. *P* values for nonneoplastic tissue versus each glioma entity. (b) An analysis on the relationship of the* TERT* promoter mutations, the expression level of TERT protein and each tumor entity by Western blotting with the most widely used, commercially available TERT antibody (sc-7215). A bar graph showed the quantification of TERT expression in each case, normalized by a loading control GAPDH expression. ^*∗*^Nonspecific bands. LE, long exposure. AA, anaplastic astrocytoma; AO, anaplastic oligodendroglioma; AOA, anaplastic oligoastrocytoma; CD, cortical dysplasia; DA, diffuse astrocytoma; GBM,* IDH*-wildtype glioblastoma; HS, hippocampal sclerosis; OA, oligoastrocytoma; OL, oligodendroglioma; IDHwt,* IDH*-wildtype diffuse astrocytoma; wt/WT, wildtype. Scale bar = 40 *μ*m.

Having confirmed the findings that all the glioma cells we analyzed showed nuclear immunostaining of TERT to various degrees, we further examined the association of the* TERT* promoter mutations with TERT immunoreactivity in our glioma samples. Unexpectedly, immunohistochemistry using TMab-6 demonstrated that immunoreactivity for TERT did not correspond to the mutational status of the* TERT* promoter region across glioma samples (Figure 4(a)). Although the staining was weak, nuclear immunoreactivity for TERT was also confirmed in both* TERT*-wildtype and mutated gliomas with the commercially available antibody for TERT (sc-7215) (Supplementary Figure  1). In addition to the hotspot mutations in the* TERT* promoter (C228T and C250T), the presence of a common SNP in the* TERT* promoter (T349C) was not associated with TERT immunoreactivity (Supplementary Table  1). TERT immunohistochemistry was not capable of identifying the differences between TERT-mutated gliomas and TERT-nonmutant gliomas (Figure 4(a)). The scores might be relevant to tumor grading and aggressiveness represented by* IDH*-wildtype GBMs and* IDH*-wildtype diffuse astrocytomas (Figure 4(a)), both of which behave aggressively with a dismal prognosis [1, 4, 14, 21]. Finally, the relationship between the* TERT* promoter mutations, the expression levels of TERT protein, and tumor entity was further verified by Western blotting (Figure 4(b)), and the level of TERT mRNA was not parallel with its mutational status across all the glioma samples (data not shown).

## 4. Discussion

The mechanism of TERT upregulation in cancer has largely remained unknown to date. Recurrent mutations at two hotspot regions (C228T and C250T) in the* TERT* promoter have been reported in various types of cancers [2, 3, 5], and a recent report outlined the mechanism that* TERT* promoter mutations reactivate TERT expression [34]. In diffuse gliomas, the sole mechanism to upregulate TERT mRNA is considered to be two hotspot mutations in the* TERT* promoter region. However, the level of TERT transcripts varies widely in expression among tumors including gliomas [5, 7], skin cancers [2, 3, 35], thyroid cancers [7], hepatocellular carcinomas [36], bladder cancers [37], and malignant lymphomas [38]. The levels of TERT mRNA tend to be increased in* TERT*-mutated tumors in comparison with* TERT*-wildtype tumors, but it is not necessarily the case for the individual tumors, indicating that mutations in the* TERT* promoter alone could not explain the wide variety in the expression of TERT transcripts. Further, to the best of our knowledge, only one report has so far investigated the expression of TERT protein in gliomas, and any difference was not detected between* TERT*-mutated and* TERT*-wildtype GBMs [7].

In the present study, we compared the expression of TERT protein with its mutational status in adult gliomas. In line with the previous studies, TERT expression was confirmed in the nuclei of oligodendroglial tumors and* IDH*-wildtype GBM which possess either of two hotspot mutations in the promoter regions of* TERT*. Surprisingly, however, even* IDH*-mutant astrocytic tumors (DA/AA) without known hotspot mutations in the* TERT* promoter displayed certain amount of TERT immunoreactivity in their nuclei. Additionally, qRT-PCR study did not demonstrate any significant correlation between* TERT* promoter mutations and the level of TERT mRNA expression. Considering our findings that immunoprecipitation with TMab-6 did demonstrate the same blotting bands as the commercially available antibody (10E9-2/sc-7215) and TMab-6 and sc-7215 showed the similar immunostaining pattern in the tumor, TERT immunoreactivity is surely increased in tumor tissue in comparison with normal tissue although the possibility of any unknown cross-reactivity cannot be completely ruled out. The finding that the TERT immunoreactivity in endothelial as well as tumor cells is strictly limited to the neoplastic tissue further supports the idea that TMab-6 detects TERT protein closely bounded to the tumor environment composed of neoplastic tissue and neovasculatures.

Our findings of an unexpected increase in the expression of TERT protein even in the* TERT*-wildtype gliomas raise the possibility that the expression of TERT mRNA and protein could be regulated by the mechanism other than its promoter mutations, including via epigenetic factors. In addition to reported hotspot mutations, certain SNPs in the* TERT* promoter region (T349C) were reported to suppress TERT transcripts [32], but we could not confirm any association of SNPs with TERT protein expression, further supporting our hypothesis that TERT expression could be epigenetically regulated.

There could be several mechanisms to increase the expression of TERT protein in* TERT*-wildtype tumors. It has been reported that DNA hypermethylation of the* TERT* promoter is associated with TERT upregulation in pediatric brain tumors [8] and other tumors [39], and histone acetylation at the* TERT* promoter regions may also be relevant to its expression [9].* IDH*-mutant diffuse astrocytic tumors often demonstrate the glioma-CpG island methylator phenotype (G-CIMP) [40, 41], and the nuclear TERT staining in diffuse astrocytomas might be epigenetically caused by hypermethylation of the* TERT* promoter [10]. Additionally, a number of human tumors maintain their telomeres by a telomerase-independent mechanism termed alternative lengthening of telomeres (ALT) caused by such abnormalities as loss of ATRX [42, 43], and this might somehow affect the expression level of TERT.

In addition to its applicability to the molecular diagnostics of gliomas, TERT could be a good therapeutic target against diffuse gliomas which are a potentially malignant, incurable brain tumor in human. A novel TERT-targeting therapy [44, 45] would be expected to specifically target* IDH*-wildtype GBM including primary brain tumor initiating cells and 1p/19q-codeleted oligodendroglioma which have characteristically possessed hotspot mutations in the* TERT* promoter region [5, 33]. Our unexpected findings suggest that a specific TERT-targeting therapy might be a promising therapeutic strategy against all types of gliomas by directly targeting tumor cells with an indirect antiangiogenic effect on tumor vasculature like the antiangiogenic agent bevacizumab, a humanized monoclonal antibody against vascular endothelial growth factor (VEGF) ligands [46], but the additional studies are essential to demonstrate that the presence of TERT protein actually implies more aggressive biology for the recommendation of treatment based on TERT immunohistochemistry.

The limitation of our study is that we have not directly measured the telomerase activity itself. Indeed, some reports demonstrated that TERT expression and its biological activity are not necessarily parallel [12]. Further, there could be the possibility that this novel antibody is picking up baseline TERT protein that may not be associated with increased telomerase efficiency or activity. It would be thus important to further validate/specify the antibody and analyze the relationship between histological TERT immunoreactivity and its enzymatic activity as a next step. Future studies are also necessary to unravel the contributing factors in upregulating the protein expression of TERT to examine if TERT protein expression could be the biomarker for the applicability of TERT-targeting therapeutics in the future.

Together, we developed a novel TERT-specific antibody, and it is sensitively and specifically applicable to immunohistochemistry for TERT in human glioma tissue. Immunohistochemistry with this TERT-specific antibody could not play the diagnostic surrogate for* TERT* promoter mutations, but we could find an unexpected increase in TERT immunoreactivity across all types of gliomas and tumor vasculature. This study is the first extensive analysis on the expression of TERT protein in human glioma tissue and suggests that TERT expression could be regulated by various mechanisms including epigenetic modifications. Future studies with the measurement of TERT activity could be useful for facilitating our understanding of telomere biology in cancer including gliomas.

## Figures and Tables

**Table 1:** Molecular information of the cases.

Classification	Number	TERT mt		TERT SNP	IDH1/2 mt	p53 IHC	ATRX IHC	1p/19q-codel
		C228T (%)	C250T (%)	T349C (%)	(%)	(%)	(%)	(%)
Astrocytic with IDH-mutant (DA/AA)	11	0	0	54.5	100	72.7	0	0
Oligodendroglial	10	70	30	30	100	0	100	100
GBM	11	45.5	18.2	90.9	0	27.3	100	0
DA/AA with IDH-wildtype	9	33.3	11.1	33.3	0	0	100	0

Total	41	36.6	14.6	53.7	51.2	26.8	73.2	24.4

AA, anaplastic astrocytoma; codel, codeletion; DA, diffuse astrocytoma; GBM, IDH-wildtype glioblastoma; IHC, immunohistochemistry; mt, mutation.

## Supplementary Materials

Supplementary Table  1 Detailed information of the cases.

Supplementary Table  2 Primer information.

Supplementary Figure  1 Immunostaining of TERT protein in human gliomas with TMab-6 and sc-7215. We immunostained TERT-wildtype and mutant gliomas with our newly developed TMab-6 and the most widely used, commercially available sc-7215. Both antibodies detected nuclear immunoreactivity of TERT in TERT-wildtype and mutated gliomas although the staining intensities were variable. AA, anaplastic astrocytoma; AO, anaplastic oligodendroglioma. Scale bar = 40 *μ*m.

Supplementary Figure  2 Immunostaining of TERT protein in human nonneoplastic brain tissue. We immunostained the cases of TERT-wildtype hippocampal sclerosis and cortical dysplasia with our newly developed TMab-6 (0.5 *μ*g/mL). Some reactive astrocytes showed nonspecific, weak cytoplasmic staining although any nuclear staining of TERT was not observed in them.
