# Supplementary material for: Elevated TERT Expression in TERT-Wildtype Adult Diffuse Gliomas: Histological Evaluation with a Novel TERT-Specific Antibody
Source: Biomed Res Int. 2018 Mar 5;2018:7945845. doi: 10.1155/2018/7945845 (PMC5859900; doi:10.1155/2018/7945845)
Supplement: Supplementary Figure 1 — Immunostaining of TERT protein in human gliomas with TMab-6 and sc-7215. We immunostained TERT-wildtype and mutant gliomas with our newly developed TMab-6 and the most widely used, commercially available sc-7215. Both antibodies detected nuclear immunoreactivity of TERT in TERT-wildtype and mutated gliomas although the staining intensities were variable. AA, anaplastic astrocytoma; AO, anaplastic oligodendroglioma. Scale bar = 40 μm. [file 7945845.f3.pptx]

## Slide 1
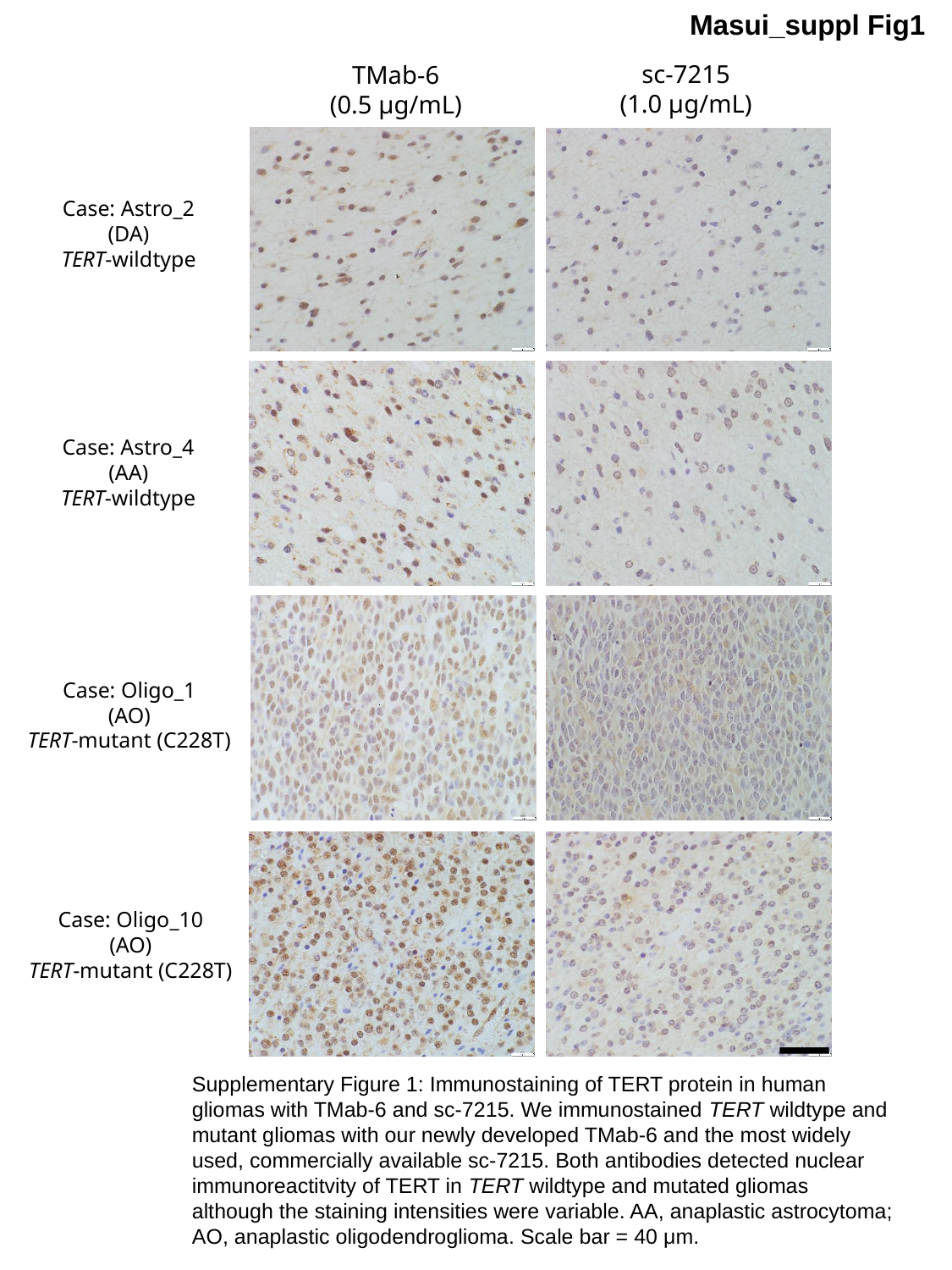

Masui_suppl Fig1
sc-7215
(1.0 μg/mL)
TMab-6
(0.5 μg/mL)
Case: Astro_2
(DA)
TERT-wildtype
Case: Astro_4
(AA)
TERT-wildtype
Case: Oligo_1
(AO)
TERT-mutant (C228T)
Case: Oligo_10
(AO)
TERT-mutant (C228T)
Supplementary Figure 1: Immunostaining of TERT protein in human gliomas with TMab-6 and sc-7215. We immunostained TERT wildtype and mutant gliomas with our newly developed TMab-6 and the most widely used, commercially available sc-7215. Both antibodies detected nuclear immunoreactitvity of TERT in TERT wildtype and mutated gliomas although the staining intensities were variable. AA, anaplastic astrocytoma; AO, anaplastic oligodendroglioma. Scale bar = 40 μm.
