# Supplementary material for: Elevated TERT Expression in TERT-Wildtype Adult Diffuse Gliomas: Histological Evaluation with a Novel TERT-Specific Antibody
Source: Biomed Res Int. 2018 Mar 5;2018:7945845. doi: 10.1155/2018/7945845 (PMC5859900; doi:10.1155/2018/7945845)
Supplement: Supplementary Figure 2 — Immunostaining of TERT protein in human nonneoplastic brain tissue. We immunostained the cases of TERT-wildtype hippocampal sclerosis and cortical dysplasia with our newly developed TMab-6 (0.5 μg/mL). Some reactive astrocytes showed nonspecific, weak cytoplasmic staining although any nuclear staining of TERT was not observed in them. [file 7945845.f4.pptx]

## Slide 1
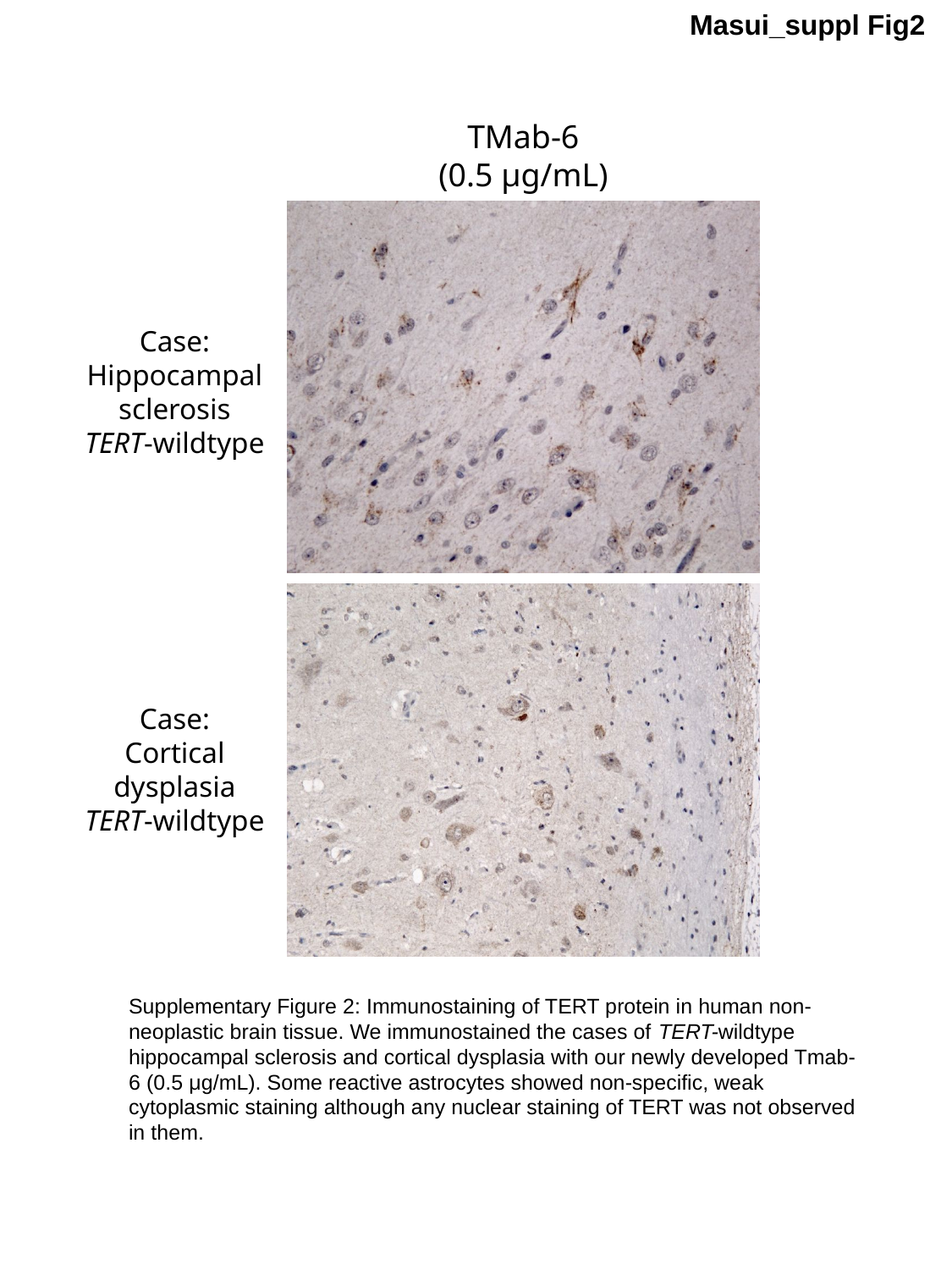

Masui_suppl Fig2
TMab-6
(0.5 μg/mL)
Case: Hippocampal sclerosis
TERT-wildtype
Case:
Cortical
dysplasia
TERT-wildtype
Supplementary Figure 2: Immunostaining of TERT protein in human non-neoplastic brain tissue. We immunostained the cases of TERT-wildtype hippocampal sclerosis and cortical dysplasia with our newly developed Tmab-6 (0.5 μg/mL). Some reactive astrocytes showed non-specific, weak cytoplasmic staining although any nuclear staining of TERT was not observed in them.
